# The Mechanism of (R,R) ZX-5 on Increasing NO Release

**DOI:** 10.3390/ijms11093323

**Published:** 2010-09-15

**Authors:** Han-Mei Xu, Jin Wei, Li Pan, Hongying Lin, Weiqiang Wang, Yihua Zhang, Zilong Shen

**Affiliations:** 1 Department of Marine Pharmacy, College of Life Science and Technology, China Pharmaceutical University, Nanjing 210009, Jiangsu Province, China; E-Mails: xuhanmei@yahoo.com.cn (H.-M.X.); tiandezhijian@126.com (J.W.); panli0225@163.com (L.P.); hongyin7719@sina.com (H.L.); wwq1979@163.com (W.W.); 2 Department of Pharmacy, University of China Pharmaceutical, Nanjing 210009, Jiangsu Province, China; E-Mail: zyhtgd@sohu.com

**Keywords:** (R,R) ZX-5, iNOS, eNOS, ERK, Akt

## Abstract

(R,R) ZX-5 has been proven to have positive effects on choroidal blood flow without affecting the sclera and ciliary bodies in New Zealand white rabbits. This study was designed to investigate the mechanisms of (R,R) ZX-5 on improving the choroidal blood flow and promoting NO production. HUVECs (human umbilical vein endothelial cells) were used to determine the production of eNOS, p-eNOS, AKT and Erk1/2 by Western blot analysis. iNOS and eNOS mRNA levels were investigated by RT-PCR and the effect of (R,R) ZX-5 on NO production were determined by eNOS activity assay. We found (R,R) ZX-5 upregulated protein expression of eNOS and iNOS, increased NO production, and reduced ERK and Akt protein level. Therefore, (R,R) ZX-5 may promote the choroidal blood flow in New Zealand white rabbits without affecting the blood flow in the iris or ciliary bodies via increasing NO production. These results suggest that (R,R) ZX-5 may function to cure and prevent Age-related macular degeneration (AMD).

## 1. Introduction

Age-related macular degeneration (AMD) is a progressive disease affecting nearly 50 million people worldwide [1,2]. It causes irreversible visual impairment and blindness which can be largely attributed to choroidal neovascularization (CNV), the development of abnormal blood vessels underneath the retina. Although CNV occurs only in 10% of AMD patients, it accounts for 90% of AMD-related blindness. The development of CNV has been linked to dysregulation of intercellular signaling molecules, such as nitric oxide (NO) [1–3], a key signaling messenger, which is synthesized in mammalian tissues by three distinct isoforms of NO synthase (NOS): neuronal NOS (nNOS), endothelial NOS (eNOS), and inducible NOS (iNOS).

Insufficient NO production due to inadequate NOS activity can lead to various eye diseases, including AMD; consistent with this, increasing NOS activity can provide NO donors to the eyes, lower the intraocular pressure, increase ocular blood flow, and relax ciliary muscle [3].

We have designed and produced more than 200 NOS regulators, and one of them, (R, R)ZX-5, has been demonstrated to promote the blood flow of choroid but not sclera and cilliary bodies in New Zealand white rabbits [4]. (R,R) ZX-5 is the *cis*-form of 1-phenyl-3-{3-methoxy-2-propoxy-5- [4-(3,4,5-trimethoxy-phenyl)-1,3 -dithiolane-2-yl]-phenyl}-thiourea). Interestingly, the *trans*-form of the compound, (S,S) ZX-5, does not display any effects on the choroidal blood flow in New Zealand white rabbit [4], indicating that the choroidal blood flow promoting effect is (R,R) ZX-5 specific. However, molecular mechanism underlining the benefical effect of (R,R) ZX-5 has not been explained clearly. In this paper, we examined the role of (R,R) ZX-5 on the expression of eNOS and iNOS, and the possible signal molecules ERK and Akt, determinant of NO production.

## 2. Materials and Methods

### 2.1. The Structure of (R,R) ZX-5

**Figure 1:**
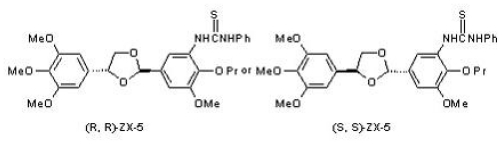
The chemical structure of ZX-5. The *cis* form, (R,R) ZX-5, is shown on the left and the transform, (S,S) ZX-5, on the right.

(R,R) ZX-5 was synthesized as described previously [4] and the structure is shown in Figure 1.

### 2.2. Cell Culture

HUVECs (human umbilical vein endothelial cells) were obtained from Nanjing Keygen biotech Co. Ltd. (Nanjing, Jiangsu province, China) and maintained at 37 °C in an incubator with 5% CO_2_. HUVECs were cultured in basal medium Dubecco’s modified Eagle’s medium (DMEM) (Gibco, Invitrogen Co., Carlsbad, CA, USA). The medium were supplement with 10% Fetal bovine serum (FBS) (Gibco, Invitrogen Co., Carlsbad, CA, USA) and 100 units/mL penicillin plus 50 μg/mL streptomycin.

### 2.3. Measurement of NO Release

Detection and quantification of NO produced by (R,R) ZX-5 treatment were conducted using the Griess assay method with the NO_2_/NO_3_ ASSAY KIT-C II (Dojindo Molecular Technologies, Inc., Rockville, MD, USA). Briefly, nitrite calibration curve and nitrate + nitrite calibration curves were first prepared, and the concentrations of nitrate and that of nitrate + nitrite in the sample were determined using the calibration curves. Finally, the nitrate concentration was calculated based on the equation:

[Nitrate]=[Nitrate+Nitrite]-[Nitrite]

### 2.4. Reverse-Transcriptase Polymerase Chain Reaction Analysis of eNOS and iNOS Expression

Reverse transcriptase polymerase chain reaction (RT-PCR) was performed using the total RNA from HUVECs cells. The primers used were 5′-CCA GCT AGC CAA AGT CAC CAT-3′ (sense) and 5′-AAA GCC TTC CGG AAG AGT CTC-3′ (antisense) for eNOS, 5′-CGT TTG ATG TCC GAG GCA-3′ (sense) and 5′-TGT GGT GAT GTC CAG GAA GTA-3′ (antisense) for iNOS, 5′-GTG AAG GTC GGA GTC AAC GGA TTT-3′ (sense) and 5′-CAC AGT CTT CTG GGT GGC AGT GAT-3′ (antisense) for GAPDH. The sizes of the RT-PCR products were 354 bp, 461 bp and 555 bp, respectively. Following electrophoresis, PCR products were analyzed using Labworks4 software (Gene Co. Ltd, HK).

### 2.5. Western Blot Analysis

For Western blot analysis, HUVECs were seeded at 3 × 10^5^ per well in 6-well culture plates and incubated for 15 h with DMEM containing 10% FBS. The cells were then treated with (R,R) ZX-5 (0, 7.5, 15 and 30 μg/mL) for 12 h. The cells were harvested with 0.05% trysin (Hyclone, UT, USA)/0.53 mM EDTA (Hyclone, UT, USA), washed in PBS, and resuspended in 100 μL of all Mammalian protein Extraction Reagent (Shanghai Generay Biotech Co. Ltd, Shanghai, China). The BCA protein assay kit (Bio-Rad, Hercules, CA, USA) was used to determine total protein concentration and purified bovine serum albumin (Sigma, NY, USA) to generate the standard curve. Concentrated protein were separated by 12% for ERK and AKT and 8% for eNOS, iNOS SDS-polyacrylamide gel electrophoresis (SDS-PAGE) and transferred to polyvinylidene difluoride (PVDF) membrane (Millipore, MA, USA). Briefly, PVDF membrane were incubated with a 1:1000 dilution of MAP kinase1/2 (ERK), AKT, eNOS, iNOS, p-eNOS Ser^1177^ and β-actin antibodies (Santa Cruz Biotechnology, CA, USA). After incubation with the appropriate secondary antibodies, blots were incubated with ECL reagents (Beyetime, Jiangsu province, China) and exposed to photographic film to detect protein expression.

### 2.6. Endothelial NO Synthase (eNOS) Activity Assay

Endothelial cells were harvested in ice-cold buffer (20 mmol/L HEPES pH 7.4, 0.2 mol/L Sucrose, 1 mmol/L EDTA, 1 mmol/L DTT, 2 mg/L Leupeptin, 2 mg/L aprotinin, 100 mg/L PMSF), and centrifuged to remove the cell debris. The supernatants were used for eNOS assay. The eNOS activity was determined as conversion of [3H] arginine to [3H] citrulline by incubating cell extracts for 10 min at 30 ºC in 10 μL (10 mM) NADPH, 0.5 μL l-[3H] arginine, 5 μL (16 mM) CaCl_2_, 1.2 μL (1 mM) arginine, and 3.3 μL ddH_2_O. Cell extracts incubated with the eNOS inhibitor, *N*G-nitro-l-arginine methylester (1 mm), served as the negative control. Converted citrulline was separated by unconverted arginine using the acidic ion exchange resin Dowex 50 W (200–400 mesh, 8% cross-linked, Sigma, St. Louis, MO, USA) as previously described [5]. Converted eNOS activity was obtained by counting per mg, per min value (cpm) of the [3H] citrulline.

### 2.7. Statistical Analysis

All values are expressed as the mean ± standard deviation (sd). Statistical differences between mean values were determined by one way ANOVA, followed by *t* test for comparison of mean values. *P* < 0.05 was considered statistically significance.

## 3. Results

### 3.1. (R,R) ZX-5 Treatment Increases NO Production in HUVECs

**Figure 2:**
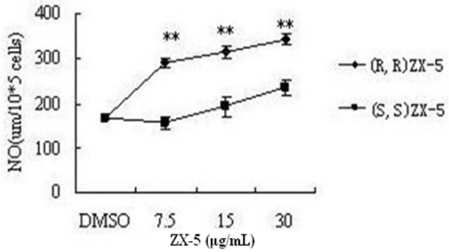
(R,R) ZX-5 enhances NO production in HUVECs: (R,R) ZX-5 significantly increasing NO production in a dose-dependent manner; (S,S) ZX-5 slightly increasing NO production in HUVECs. Data are presented as mean ± sd of 2 experiments. **P ≤ 0.01, compared with control cells.

In the rabbit, (R,R) ZX-5 treatment released a significant amount of NO at concentrations of 7.5, 15, and 30 μg/mL, and increased choroidal blood flow when 50 μL of 1% (R,R) ZX-5 solution was instilled into eyes. However, no such effect of the compound was observed in the blood flow of the iris or ciliary body [4]. The same results were observed in human endothelial cells. At 15 h following (R,R) ZX-5 treatment, NO production in HUVECs was increased in a dose-dependent manner (Figure 2).

### 3.2. (R,R) ZX-5 Up-Regulates iNOS mRNA in a Dose-Dependent Manner

**Figure 3:**
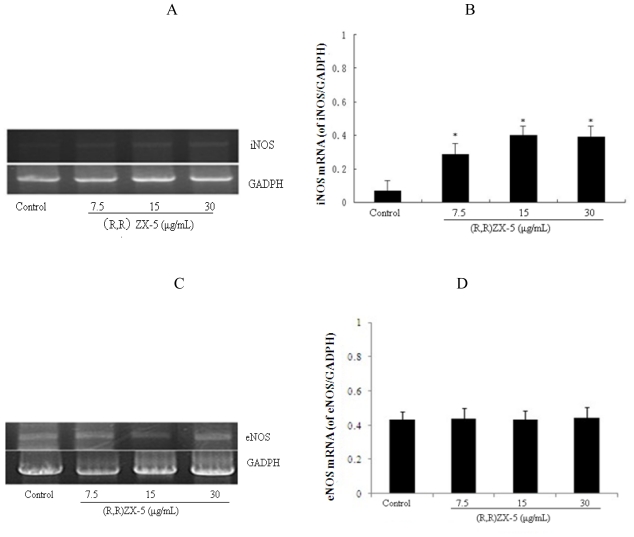
Analysis of iNOS and eNOS mRNA levels in HUVECs after treatment with 0, 7.5, 15, or 30 μg/mL(R,R) ZX-5 for 15 h. **A** and **B:** (R,R) ZX-5–induced iNOS transcription in HUVECs (of iNOS/GADPH). **C** and **D:** (R,R) ZX-5–induced eNOS transcription in HUVECs (of eNOS/GADPH). A and C are Western blots. In B and D, columns represent mean ± sd of experiments shown in A and C. *P ≤ 0.05 compared with control cells, n = 6.

To test whether (R,R) ZX-5 increased NO production in response to iNOS and eNOS expression, we analyzed iNOS and eNOS mRNA levels in HUVEC cells in the presence or absence of (R,R) ZX-5 using an RT-PCR assay. We found that (R,R) ZX-5 enhanced iNOS mRNA level in a concentration-dependent manner (Figure 3A, B) and had no effect on eNOS mRNA (Figure 3C, D) in HUVEC cells. Compared with DMSO stimulated control cells, cells treated with 30 μg/mL (R,R) ZX-5 for 15 hours displayed a significant upregulation of iNOS mRNA of approximately 258% (Figure 3B).

### 3.3. (R,R) ZX-5 Treatment Leads to Increased iNOS and eNOS Protein Expression

**Figure 4:**
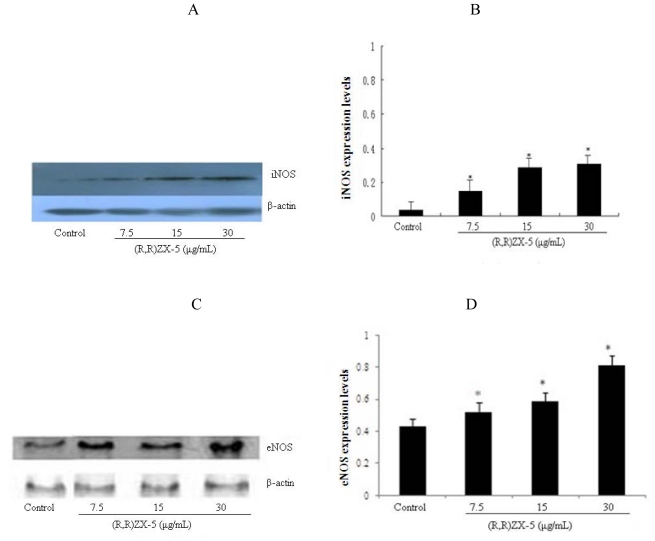

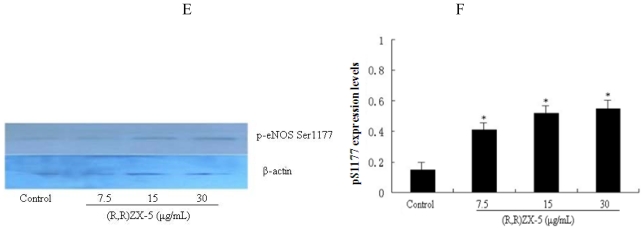
Western blot analysis of (R,R) ZX-5-induced iNOS and eNOS protein expression. **A**, **C**, **E**: protein expression of iNOS, eNOS and p-eNOS Ser^1177^ in HUVECs; **B**, **D**, **F**: effects of (R,R) ZX-5 (0, 7.5, 15, 30 μg/mL for 15 hours)–induced iNOS, eNOS (of NOS/β-actin) and p-eNOS Ser^1177^ ((of p-eNOS Ser^1177^/total eNOS) protein expression in HUVECs. Columns represent mean ± sd of the experiments, *P ≤ 0.05 compared with control cells, *n* = 6.

The iNOS and eNOS protein levels in (R,R) ZX-5-treated HUVEC cells were also analyzed and compared with those in DMSO stimulated cells. In DMSO-stimulated control cells, the production of eNOS protein was not affected. However, (R,R) ZX-5 treatment for 15 h induced a significant up-regulation of iNOS and eNOS protein (Figure 4 A and C), and (R,R) ZX-5 treatment for 3 h also induced a significant up-regulation of p-eNOS Ser^1177^ protein (Figure 4E). HUVEC cells treated with 30 μg/mL (R,R) ZX-5 exhibited a 80%, 62% and 71% increase in the iNOS, eNOS and p-eNOS Ser^1177^ protein level (Figure 4B, D, and F), respectively.

### 3.4. (R,R) ZX-5 Treatment Inhibits ERK and AKT Expression

**Figure 5:**
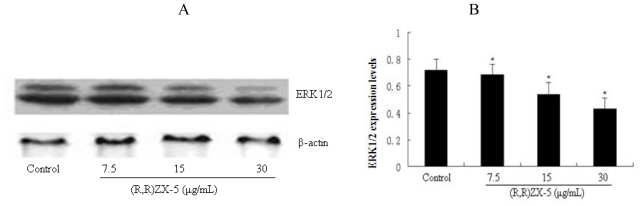

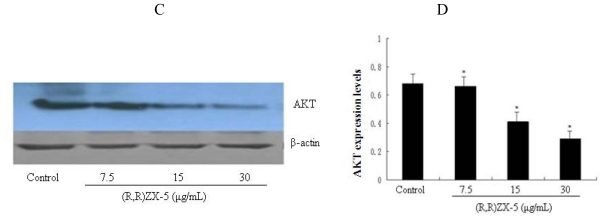
Western blot analysis of ERK and Akt protein expression levels in HUVECs. (**A**, **C**): Western blots showing protein expression of ERK and AKT in HUVECs, respectively; (**B**, **D**) the effect of (R,R) ZX-5 (0, 7.5, 15, 30 μg/mL for 15 h) on ERK and Akt protein expression in HUVECs, respectively, (of samples/β-actin). *P ≤ 0.05 compared with control cells, *n* = 6.

HUVEC cells were treated with various doses of (R,R) ZX-5 (0, 7.5, 15 and 30 μg/mL) for 15 h, followed by Western blot analysis of ERK and AKT from the cell lysates with anti-ERK and AKT monoclonal antibodies. As shown in Figure 5A and B, 30 μg/mL (R,R) ZX-5 treatment caused a significant decrease of ERK at the protein level (percentage decreases were 2.3%, 9.5% and 34.2% at 7.5, 15 and 30 μg/mL (R,R) ZX-5, respectively). Notably, (R,R) ZX-5 reduced ERK1/2 protein expression in a dose-dependent manner. As shown in Figure 5C and D, (R,R) ZX-5 from 15 μg/mL treatment caused a significant decrease of AKT at the protein level (inhibition percentages were 1.2%, 31.7% and 43.8% at 7.5, 15 and 30 μg/mL (R,R) ZX-5, respectively).

### 3.5. (R,R) ZX-5 Enhances eNOS Activity in HUVECs

**Figure 6:**
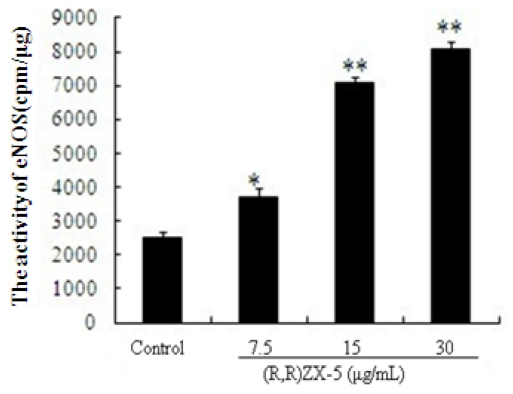
The increased activity of eNOS after (R,R) ZX-5 treatment for 15 h. Columns represent mean ± sd of the experiment, *P ≤ 0.05, **P ≤ 0.01, compared with control cells, *n* = 6.

We further analyzed the total eNOS activity in HUVECs cells treated with DMSO, 7.5, 15, 30 μg/mL (R,R) ZX-5 for 15 h. The eNOS activity assay was performed in the presence of [H^3^] L-Cit. As seen in Figure 6, in (R,R) ZX-5 treated cells, the eNOS activity was significantly higher than that in the control. (R,R) ZX-5 enhanced eNOS activity in a concentration-dependent manner in HUVECs cells. Compared to the control, cells incubated with 30 μg/mL (R,R) ZX-5 for 15 h showed an increase in eNOS activity by 325%.

## 4. Discussion

Our prior study has shown that (R,R) ZX-5 promotes the release of NO and specifically increases choroidal blood flow in New Zealand white rabbit without affecting the blood flow in the iris or ciliary body [4], suggesting that (R,R) ZX-5 may be used for the prevention and treatment of AMD in elderly people.

NO can be produced by three isoforms of NO synthase (NOS): endothelial NOS (eNOS), neural NOS, and inducible NOS (iNOS). Although NO over-production by iNOS can be toxic, a low level of NO produced by constitutively expressed eNOS is necessary to maintain the normal endothelial function [6]. Thus, eNOS and iNOS appear to be attractive candidate genes of AMD susceptibility for analysis.

Based on the structure, (R,R) ZX-5 is not a substrate of NOS. Thus, NOS cannot directly generate NO from (R,R) ZX-5. Instead, we show here that (R,R) ZX-5-enhanced NO production in HUVEC cells is via increased eNOS activity that results from up-regulation of eNOS expression at the protein level, also via increased iNOS expression at both the mRNA and protein levels. Activity of eNOS is known to be mainly regulated at the post-translational levels in a complex fashion by acylation, protein-protein interactions, intracellular trafficking, and phosphorylation. Signaling pathways that regulate eNOS activity include phosphoinositide 3-kinase/Akt, cyclic nucleotide-dependent kinases [PKA (protein kinase A) and PKG], PKC, as well as ERKs (extracellular-signal-regulated kinases) [7].

Mitogen-activated protein kinases (MAPKs) have been implicated in the regulation of NO production. However, the roles of ERK in eNOS regulation remain controversial. While inhibition of ERK signaling has been shown to attenuate eNOS activity stimulated by a variety of factors in several cell lines [8–12], it can also cause little change [13–16] or even an increase in eNOS activity [7,17]. Similarly, activated ERK phosphorylates eNOS in bovine aortic endothelial cells, leading to reduced eNOS enzyme activity [18,19]. By contrast, our results showed that (R,R) ZX-5 simultaneously up-regulates eNOS protein activity and inhibiting ERK protein expression. The research indicates ERK might be involved in the regulation of eNOS activity by (R,R) ZX-5, further study needs to be done to identify the relationship between the expression and the activity of ERK and (R,R) ZX-5-induced eNOS activation.

It is tempting to speculate that, upon (R,R) ZX-5 treatment, reduced ERK may lead to induced phosphorylation of eNOS, leading to enhanced eNOS activity and production of more NO in the cells. This proposed mechanism can be supported from the observation by Bernier *et al.* that ERK may directly phosphorylate eNOS *in vitro*, and that eNOS activity is enhanced by MEK/ERK inhibition in ATP-stimulated HUVECs [17]. Recently, enhanced eNOS activity in cells after MEK/ERK inhibition has also been reported by other researchers [7,19]. The present study proved that (R,R) ZX-5 affect eNOS protein expression and activity in an indirect manner by inhibiting ERK and thereby phosphorylating eNOS at alternative residues.

Choroidal neovascularization (CNV) or choroidal angiogenesis is the creation of new blood vessels in the choroid layer of the eye; and this is a common symptom of the degenerative maculopathy wet AMD. It was found that the formation of CNV might have been inhibited or stopped by downregulation of the activity of the crucial key downstream effectors, Akt and ERK, of the PI3K/Akt and MEK/ERK pathway [20–23]. In our study, we found that (R,R) ZX-5 markedly downregulated the protein levels of Akt and ERK. These data suggest (R,R) ZX-5 may stop and inhibits the formation of CNV.

In conclusion, the data presented here provide the first evidence that(R,R) ZX-5 has positive effects on NOS (eNOS and iNOS) and choroidal blood flow via the MEK/ERK- and PI3K/Akt -dependent signaling pathways. Therefore, our data will provide a potential role for (R,R) ZX-5 in the pathogenesis of diseases associated with AMD and will contribute to the development of new therapeutic strategies for AMD.
